# MULTIPLE PERSPECTIVES ON WHAT (IF ANY) IS AN OPTIMAL TIME FOR PEOPLE WITH DEMENTIA TO MOVE TO A CARE HOME

**DOI:** 10.1093/geroni/igz038.438

**Published:** 2019-11-08

**Authors:** Kritika Samsi, Laura Cole, Jill Manthorpe

**Affiliations:** King’s College London, London, United Kingdom

## Abstract

Deciding an ‘optimal’ time for a person with dementia to move to a care home may be difficult for people with dementia, family carers, and professionals who support them; but there is currently limited evidence to help make this decision. Using phenomenology, we carried out qualitative interviews with 20 family carers, 5 people with dementia, 20 care home managers and 20 social workers, about their experiences, views and attitudes regarding timing of a move to a care home. Social workers indicated that managing risks and safety of person with dementia living in their own home were paramount when considering where person with dementia should live. These concerns included mishandling gas and electrical equipment at home, wandering and getting lost outside, and breakdown of family care. They and care home managers valued wishes of the person with dementia, and minimising any emotional distress to them when a move did come about. Family carers reported feeling stressed, and guilty around decision-making and ultimate move of their relative to a care home. Many described weighing up various risks when reaching ‘tipping point’ and making trade-offs between available options or uncertain future choices. Participants with dementia recognised they had struggled to cope at home and needed more support; however, many found the move difficult as they relocated nearer to family, away from their home and friends, and resigned themselves to less independence. Most people with dementia reported that their carers initiated discussions about timing of move, and that family discussions about this were common.

